# CDetection.v2: One-pot assay for the detection of SARS-CoV-2

**DOI:** 10.3389/fmicb.2023.1158163

**Published:** 2023-03-23

**Authors:** Xinge Wang, Yangcan Chen, Xuejia Cheng, Si-Qi Wang, Yanping Hu, Yingmei Feng, Ronghua Jin, Kangping Zhou, Ti Liu, Jianxing Wang, Kai Pan, Bing Liu, Jie Xiang, Yanping Wang, Qi Zhou, Ying Zhang, Weiye Pan, Wei Li

**Affiliations:** ^1^State Key Laboratory of Stem Cell and Reproductive Biology, Chinese Academy of Sciences, Institute of Zoology, Beijing, China; ^2^Bejing Institute for Stem Cell and Regenerative Medicine, Beijing, China; ^3^Chinese Academy of Sciences, Institute for Stem Cell and Regenerative Medicine, Beijing, China; ^4^Savaid Medical School, University of Chinese Academy of Sciences, Beijing, China; ^5^Beijing SynsorBio Technology Co., Ltd., Beijing, China; ^6^Department of Science and Technology, Beijing Youan Hospital, Capital Medical University, Beijing, China; ^7^Beijing Ditan Hospital, Capital Medical University, Beijing, China; ^8^Hubei Provincial Center for Disease Control and Prevention, Wuhan, China; ^9^Shandong Center for Disease Control and Prevention, Jinan, China; ^10^Tonghua Central Hospital, Tonghua, Jilin, China; ^11^Tongji Medical College of Huazhang, Wuhan Jinyintan Hospital, University of Science and Technology, Wuhan, China; ^12^Hubei Provincial Hospital of Traditional Chinese Medicine, Wuhan, China

**Keywords:** CRISPR/Cas12b, SARS-CoV-2, molecular diagnosis, one-pot detection platform, clinical samples

## Abstract

**Introduction:**

The ongoing 2019 coronavirus disease pandemic (COVID-19), caused by severe acute respiratory syndrome coronavirus-2 (SARS-CoV-2) and its variants, is a global public health threat. Early diagnosis and identification of SARS-CoV-2 and its variants plays a critical role in COVID-19 prevention and control. Currently, the most widely used technique to detect SARS-CoV-2 is quantitative reverse transcription real-time quantitative PCR (RT-qPCR), which takes nearly 1 hour and should be performed by experienced personnel to ensure the accuracy of results. Therefore, the development of a nucleic acid detection kit with higher sensitivity, faster detection and greater accuracy is important.

**Methods:**

Here, we optimized the system components and reaction conditions of our previous detection approach by using RT-RAA and Cas12b.

**Results:**

We developed a Cas12b-assisted one-pot detection platform (CDetection.v2) that allows rapid detection of SARS-CoV-2 in 30 minutes. This platform was able to detect up to 5,000 copies/ml of SARS-CoV-2 without cross-reactivity with other viruses. Moreover, the sensitivity of this CRISPR system was comparable to that of RT-qPCR when tested on 120 clinical samples.

**Discussion:**

The CDetection.v2 provides a novel one-pot detection approach based on the integration of RT-RAA and CRISPR/Cas12b for detecting SARS-CoV-2 and screening of large-scale clinical samples, offering a more efficient strategy for detecting various types of viruses.

## 1. Introduction

Since the outbreak of the COVID-19 pandemic, an effective strategy to prevent the spread of COVID-19 is to conduct the large-scale nucleic acid screening, followed by separating persons with confirmed or suspected COVID-19 infection from individuals who tested negative (Drain, 2022; Peeling et al., 2022). Nucleic acid detection, which relies on techniques such as sequencing, PCR, real-time quantitative PCR (RT-qPCR), and molecular switch design (Mohamadian et al., 2021; Yoo et al., 2021), is a crucial diagnostic tool to identify the viral infection at early stage. Isothermal amplification also plays an important role in COVID-19 screening due to its portability and reasonable consultation time (Yan et al., 2014; Zhao et al., 2015). However, the specificity of isothermal amplification is compromised by non-specific amplification (Schneider et al., 2019; Kaminski et al., 2021). Therefore, development of a detection platform with higher sensitivity, faster speed and greater accuracy is important.

The CRISPR/Cas system is part of the microbial adaptive immune system and is comprised of the CRISPR array and CRISPR-associated proteins (Cas) (Zhang, 2019). Since the *trans*-cleavage activity of Cas enzymes can specifically target a certain region of double strand DNA under the guidance of sgRNA (Joung et al., 2020), single CRISPR-Cas (such as Cas12 and Cas13) effector-mediated DNA *trans*-cleavage following isothermal amplification, has been used to develop portable nucleic acid detection systems with single-base resolution and high-sensitivity, such as the SHERLOCK and DETECTR technologies (Gootenberg et al., 2017, 2018; Chen et al., 2018). Despite the increased specificity of this two-step approach, its application in large-scale screening is still hampered because it does not shorten the processing time compared with isothermal amplification alone (Li et al., 2019; Guo et al., 2020). Attempts have been made to integrate amplification and CRISPR-based detection into a one-pot system that could reduce the risk of aerosol contamination caused by repeated capping and frequent exposure to the environment, thus improving portability (Huang et al., 2020; Wang et al., 2022). However, the sensitivity of these one-pot systems was reduced due to the lack of a compatible system for the isothermal amplification and CRISPR to work simultaneously (Guo et al., 2020; Joung et al., 2020). Given the high risk of COVID-19 contagion, development of a one-pot CRISPR detection system with high sensitivity, high specificity and rapid detection is necessary to control the pandemic and reduce the burden on society. In addition, these platforms can be used for portable detection in conjunction with lateral flow assays (Ding et al., 2020; Joung et al., 2020; Nguyen et al., 2020; Lu et al., 2022).

In our previous work, we developed a two-step detection system with high-sensitivity using CRISPR/Cas12b whose *trans*-cleavage activity is efficient, and enables sensitive detection at single-base resolution (Teng et al., 2018, 2019). However, this two-step detection is still complicated and time-consuming (Guo et al., 2020), prompting us to develop a one-pot approach for clinical detection by optimizing the components of the reaction system and the detection procedure. In this study, we developed a Cas12b-based one-pot platform by integrating isothermal amplification and CRISPR detection into one step, called CDetection.v2, which enables the rapid detection of SARS-CoV-2 in 30 min without compromising the sensitivity and accuracy of detection.

## 2. Materials and methods

### 2.1. Oligo preparation and synthesis

All conventional primers used in the experiments were synthesized by Sangon (Shanghai, China). RT-RAA primers were purified by high performance liquid chromatography (HPLC). The modified FAM and BHQ1 reporters were synthesized by Genscript (Nanjing, China). Standard original SARS-CoV-2 nucleic acid samples [Beijing, GBW(E)091089] and Delta (Beijing, GBW09316), Omicron (Beijing, GBW09318) variants standard nucleic acid samples were all obtained from the National Institute of Metrology. Guide RNA and RdRp RNA were generated by *in vitro* transcription using the HiScribe™ T7 Quick High Yield RNA Synthesis Kit (NEB, Ipswich, England, E2050S) according to the manufacturer’s instructions. After the transcribed DNA template was digested by DNase I (RNase-free) (NEB, Ipswich, England, M0303S), the sgRNA was purified with the Monarch^®^ RNA Cleanup Kit (NEB, Ipswich, England, T2040S) and quantified using Nanodrop. Purified sgRNA can be assessed through agarose electrophoresis and stored at −80°C.

### 2.2. Protein purification

The Cas12b protein was produced and purified by Genscript (Nanjing, China). The expression plasmid BPK2014-AaCas12b (Addgene, 121949) (Teng et al., 2018) was transformed into Chemically Competent BL21 (DE3) Cells (Transgene, Beijing, China, CD601-02), the bacteria were transferred to fresh LB medium with chloramphenicol and grown at 37°C until the OD_600_ was 0.6. Protein expression was induced for 16 h at 16°C. The bacteria were centrifuged and resuspended in lysis buffer (10 mM Tris-HCl, 200 mM NaCl, 10 mM Imidazole, 1 mM DTT, pH7.5). After sonication, the supernatant was passed through a 0.22 filter (Millipore, Burlington, MA, United States, SLGP033RB) and the resultant filtrate was then incubated with Ni-NTA agarose (Thermo Fisher, Waltham, MA, United States, R90115) at a temperature of 4°C for a period of 1 h. The protein was purified and washed with an imidazole gradient (included 10, 20, 50 and 100 mM imidazole). The protein was then eluted with 500 mM imidazole and subsequently desalted in the dialysate after being passed through the dialysis bag (Solarbio, Beijing, China, YA1046). The protein is ultimately concentrated utilizing Amicon^®^ Ultra-4 Centrifugal Filter Unit (Millipore, Burlington, MA, United States, UFC8050). The concentration of the purified proteins was determined by Pierce™ BCA Protein Assay Kit (Thermo Fisher, Waltham, MA, United States, 23225). Purified protein be stored at −80°C.

### 2.3. Cas12b-based *trans*-cleavage assays

The Cas12b assay was performed in the presence of 50 nM AapCas12b, 30 nM sgRNA, 40 nM activator, 200 nM ssDNA FQ reporter and NEBuffer™ 2 (NEB, Ipswich, England, B7002S) in a 16 μL reaction. The activator is prepared by utilizing the synthesized 100 bp ssDNA as a template and refining the product obtained from PCR with Q5^®^ High-Fidelity 2X Master Mix (NEB, Ipswich, England, M0492S). The concentration of activator is determined by Nanodrop, and this is subsequently used to calculate the concentration in relation to the molecular weight. Reactions were incubated with a temperature gradient for the indicated time in an Applied Biosystems Veriti™ Thermal Cycler. The reaction was then transferred to a Corning^®^ 384-well Polystyrene NBS Microplate (Corning, 3571) and detection was performed in a multi-detection microplate reader (BioTek Synergy 4) in the fluorescence mode (λex = 485 nm; λem = 528 nm, transmission gain = 61). The relative fluorescence unit can be analyzed directly.

### 2.4. CDetection.v2 assays

CDetection.v2 assays were performed by using commercial freeze-dried RT-RAA (Hangzhou ZC Bio-Sci&Tech Co., Ltd., S003ZC), according to the manufacturer’s instructions. The assay system was prepared in advance and divided into three components. A total of 35 μl component A contained RT-RAA reaction buffer, 400 nM RT-RAA primer, 1200 nM FAM–BHQ1 fluorescent probe and 14 mM magnesium acetate final concentration in the final 50 μl system. Component B is the RT-RAA dry powder purchased from the manufacturer. A total of 5 μl component C consists of 200 nM sgRNA, 50 nM Cas12b protein, 200 UI RNase Inhibitor (Promega, Wisconsin, MI, United States, N2515), 50 mM NaCl, 2 mM HEPES (PH 7.4), 3 mM DTT, 10 nM EDTA and 0.001% Tween final concentration in the final 50 μl system. To perform the assay, 10 μl of the sample were added to component B, dissolved the dry powder B using the sample. Then, component A should be included to guarantee the stability of the proteins related to the amplification process in the system, followed by sequential addition of component C and gentle vortexing. It is important to pay attention to the reaction system and ensure that the sample is adequately mixed, yet not too vigorously to avoid impacting the function of enzyme. Subsequently, the only step left is to initiate the instrument to run the program for detection. The reaction was first performed at 42°C for nucleic acid template amplification for 7 min and then the temperature was increased to 52°C for *trans*-cleavage. The reaction volume was 50 μl, and the assay was performed in the Applied Biosystems QuantStudio 6 real-time PCR instrument. Rn values were exported and analyzed using GraphPad Prism 8 software.

### 2.5. Positive reference value determination of CDetection.v2

In three research institutions, five clinical samples were selected for throat swabs to determine the positive judgment value. Using two batches of reagents, 15 positive samples were diluted to 1 × 10^4^ copies/ml and tested for 5 consecutive days. Each sample was tested once per day. Values obtained from each batch were analyzed for normality using SPSS 20.0 analysis software. The results showed that the data did not support the null hypothesis of normality at 5% significance level (*p* = 0.018). The results of the Kolmogorov–Smirnov test showed that the distribution of marginally positive samples was not normal. For the following calculations, a non-parametric method was used to determine the positive judgment value in the subsequent calculations, assuming a tolerable false-negative error rate of 1%.

So, rank position (1%) = 0.5 + 75 × 0.01 = 1.25

LoB = {X (1) + 0.25 × [X (2) − X (1)]}

LoB (Cutoff A) = X (0.248) + 0.25 × [X (0.254)−X (0.248)] = 0.2495

LoB (Cutoff B) = X (0.247) + 0.25 × [X (0.259)−X (0.247)] = 0.2500

To summarize, the larger data value of the two batches was chosen as the positive judgment value, so the positive judgment value must be ≥ 0.250.

Non-parametric approaches were used to ascertain reference intervals for negative samples as well. Setting the maximum acceptable false positive error rate at 1%.

Rank position (99%) = 0.5+72 × 0.99 = 71.78

LoB = {X (71) + 0.78 × [X (72)−X (71)]}

LoB (Cutoff A) = X (0.236) + 0.78 × [X (0.241)−X (0.236)] = 0.2399

LoB (Cutoff B) = X (0.238) + 0.78 × [X (0.238)−X (0.236)] = 0.2396

Thus, the negative judgment value must be < 0.24.

### 2.6. Analysis of clinical samples by RT-qPCR

Clinical samples were collected from the Tonghua (Jilin, China), Linyi (Shandong, China) and Wuhan (Hubei, China) CDC (Centers for disease control and prevention). Information of clinical samples are provided in the Supplementary material. Throat swabs of COVID-19 patients or suspected patients are used to acquire samples for the purpose of diagnosis, which are extracted with the nucleic acid extraction or purification kit (Guangzhou Magen Biotechnology Co., Ltd, IVD5412). The Ct values of the clinical samples were measured using HiScript^®^ II U+ One Step qRT-PCR Probe Kit according to the manufacturer’s instruction (Vazyme, Q223-01). Add 5 μl sample into 25 μl enzyme master mix. A 30 μl reaction included buffers, Rox, 1.5 μl One step U+ Enzyme Mix, 200 nM RdRP_SARSr-F3, 200 nM RdRP_SARSr-F3 RdRP_SARSr-R2 and 100 nM RdRP_SARSr-P2. The reaction was performed using the 7500 Fast Real-Time PCR System (Applied Biosystems) as following procedure: (1) reverse transcription at 55°C for 15 min; (2) a pre-denaturation step at 95°C for 30 s; (3) 10 s at 95°C for and 30 s at 60°C for 45 cycles.

### 2.7. The analysis of primers and sgRNA sequences

The SARS-CoV-2 sequence was downloaded from the virus database in NCBI.^1^ We conducted a random sampling of a specific number of virus sequences from the database by country, including multiple variants. Random sequences of no more than 100 samples were selected from each country. We compared these sequences with the RT-RAA primers and the 20 nt spacer of the sgRNA that we utilized. The software package BLAST+2.13.0^2^ was used for comparison and analysis. The samples were classified by region and time. The ratio of mismatched sequences to the total number of sequences in the region will be calculated in order to evaluate the tolerance of the primers and sgRNA of the detection system. The comparison results were analyzed using GraphPad Prism 8.

### 2.8. Statistical analysis

All experiments were performed in at least triplicate. Statistical analysis was carried out using GraphPad Prism 8.

## 3. Results

### 3.1. Establishment of CDetection.v2 platform

**FIGURE 1:**
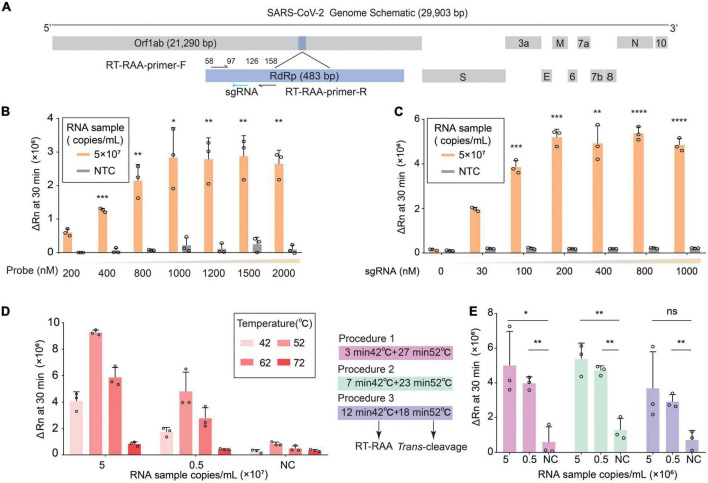
Establishment of CDetection.v2 platform. **(A)** Schematic diagram of the SARS-CoV-2 genome. RdRp segment is marked in light blue. RT-RAA primers are indicated by black arrows and sgRNAs are indicated by blue arrows. **(B)** Detection efficiency of CDetection.v2 under different concentration of ssDNA reporter. The efficiency is evaluated by delta Rn measured by real-time quantitative PCR (RT-qPCR) instrument. The delta Rn value measured at 30 min is plotted on a histogram. Error bars represent standard deviation (SD) of mean, *n* = 3 replicates. Two-tailed, unpaired Student’s *t*-test, *p < 0.05, **p < 0.01, ***p < 0.001. **(C)** Detection efficiency of CDetection.v2 under different concentration of sgRNA. The efficiency is evaluated by delta Rn measured by RT-qPCR instrument, error bars represent SD of mean, *n* = 3. Two-tailed, unpaired Student’s *t*-test, **p < 0.01, ***p < 0.001, ****p < 0.0001. **(D)** Detection efficiency of CDetection.v2 under different combinations of temperatures and concentration of RNA targets. The efficiency is evaluated by delta Rn measured by RT-qPCR instrument. Different temperatures are indicated by red bars of different sizes. Error bars represent SD of mean, *n* = 3. **(E)** The analysis of the detection time ratio using two-temperature-stage procedure. The 30-minute incubation period has been divided into two stages, the amplification stage and the *trans*-cleavage stage, each with a specific temperature. These two temperature stages can be combined in different ratios to create three different conditions. The changes in delta Rn values of the RNA samples can be monitored under these three different conditions. Error bars represent SD of mean, *n* = 3. Two-tailed, unpaired Student’s *t*-test, ns: not significant, *p < 0.05, **p < 0.01.

To establish a Cas12b-based SARS-CoV-2 one-pot detection platform, we selected the conserved sequence RdRp (RNA-dependent RNA polymerase) and the RT-RAA (reverse transcription recombinase-aided amplification) primers from our previous publication on Orf1ab (Guo et al., 2020), where RdRp was the target and RT-RAA primers were used for isothermal amplification (Figure 1A). Instead of using the previous two-step approach (Guo et al., 2020), RT-RAA and AapCas12b (Cas12b for short) were added to a single tube allowing simultaneous amplification and detection of SARS-CoV-2 in a single reaction. Because integrating of amplification and *trans*-cleavage in one reaction resulted in decreased fluorescence intensity (Guo et al., 2020), we first optimized the concentration of fluorescent probe in the reaction. The results showed that increasing the concentration of the fluorescent probe enhanced the fluorescent intensity when detecting standard RNA samples with different copy numbers (Figure 1B). As our previous two-step approach showed that increased sgRNA enhanced enzyme catalysis in a dose-dependent manner (Guo et al., 2020), we increased the concentration of sgRNA to improve the performance of one-pot detection assay. The results showed that a strong fluorescence signal was detected when the sgRNA concentration was increased to 200 nM (Figure 1C). Furthermore, we focused on increasing Cas protein *trans*-cleavage activity by optimizing the temperature because Cas12b is thermally stable and can perform extensive *trans*-cleavage at high temperature (Teng et al., 2018; Joung et al., 2020). We first examined the *trans*-cleavage activity of Cas12b in response to dsDNA activators at different temperatures and found that Cas12b exhibited a robust *trans*-cleavage activity from 25C to 65°C (Supplementary Figure 1). Then we tested the suitable temperature of RT-RAA from 25 to 65°C for establishing one-step reaction, while the recommended temperature for pre-amplification was 42°C. Since the components of the RT-TAA reaction were changed in one-pot system, which might affect the molecules movement and Cas enzymatic kinetics caused by increased temperature, we tested the optimal temperature for *trans*-cleavage at 42, 52, 62 and 72°C for 23 min, respectively. The result showed that 52°C was the best temperature for Cas12b-catalyzed ssDNA-cleavage (Figure 1D). To further increase the sensitivity, time ratio of isothermal amplification to *trans*-cleavage within 30 min was tested. Three different time ratios were evaluated and found that amplification for 7 min followed by *trans*-cleavage for 23 min gave a robust fluorescence signal. Notably, the sensitivity of RNA detection showed non-significantly improved when we prolonged the detection time (Figure 1E and Supplementary Figure 2). Collectively, our findings demonstrate that the CRISPR/Cas12b *trans*-cleavage activity and detection sensitivity can be improved by increasing the concentration of reactants, raising the reaction temperature, and optimizing the reaction process. These results show the successful establishment of one-pot detection system for SARS-CoV-2 based on RT-RAA and Cas12b with highly sensitive and rapid speed, named as CDetection.v2.

### 3.2. Evaluation of the detection capabilities of CDetection.v2

**FIGURE 2:**
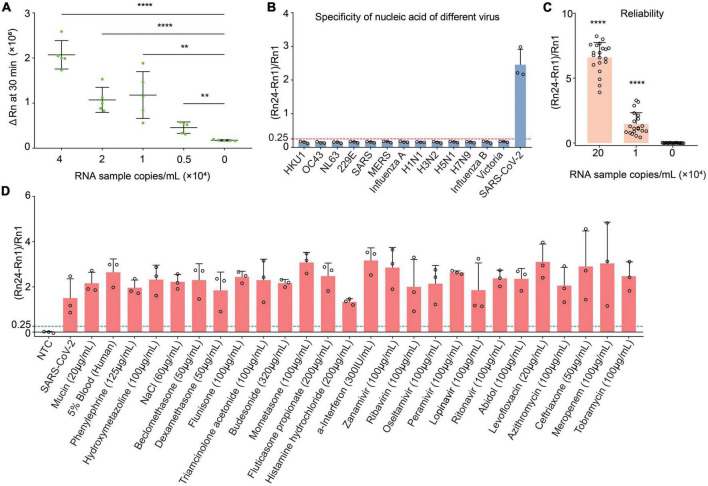
Evaluation of the detection capabilities of CDetection.v2. **(A)** The sensitivity of CDetection.v2 on gradient diluted RNA samples. The sensitivity was evaluated by delta Rn measured by real-time quantitative PCR (RT-qPCR) instrument. Error bars represent SD of mean, *n* = 5 biological replicates. Two-tailed, unpaired Student’s *t*-test, **p < 0.01, ****p < 0.0001. **(B)** Evaluation of cross reactivity of CDetection.v2. The degree of separation of nucleic acid samples containing target from 8 human epidemic viruses are serially diluted. Error bars represent SD of mean, *n* = 3. **(C)** Repeatability analysis of CDetection.v2 assay. Error bars represent SD of mean, *n* = 20 biological replicates. Two-tailed, unpaired Student’s *t*-test, ****p < 0.0001. **(D)** Influence of internal and external interfering substances on CDetection.v2. The potential interferences detected include the original substances in the sample and the substances introduced during sample collection and preparation, which are mixed into the unextracted samples for interference simulation. Error bars represent SD of mean, *n* = 3.

The sensitivity of CDetection.v2 platform was determined by detecting serial diluted standard RNA sample with designated copy number. Results showed that, compared with negative samples, the fluorescent intensity was significantly increased when the concentration of RNA sample reached to 5,000 copies/ml. Moreover, increased concentration of RNA samples produced enhanced fluorescent intensity. Compared with 1 × 10^10^ copies/ml sensitivity of one-pot assays from our previous publication (Guo et al., 2020), this platform exhibited a very high sensitivity and was able to detect 5,000 copies/ml RNA template within 30 min (Figure 2A).

In order to eliminate the influence of the initial well fluorescence on the results, the (Rn-R1)/R1 was chosen as the interpretation of the fluorescence detection results. Meanwhile, to further verify the effectiveness of this system, diluted clinical SARS-CoV-2 samples with designated copy number were used and the (Rn24-Rn1)/Rn1 value was used as the readout of CDetection.v2. To more accurately interpret and quantify the detection results, the readout standard of CDetection.v2 was first determined. Fifteen clinical SARS-CoV-2 samples were randomly selected, diluted into 1 × 10^4^ copies/ml and analyzed at five different time points. Based on the results of these fifteen samples, models were formulated to fit the data (Supplementary Figure 3). Based on the requirements of “*Evaluation of Detection Capability for Clinical Laboratory Measurement Procedures, 2nd Edition*,” a false positive error rate of 1% was considered as acceptable, while the sample with a value of (Rn24-Rn1)/Rn1 greater than 0.25 was considered as positive.

In order to gain a more comprehensive insight into the performance of the detection system when the form of result interpretation was clarified, it is essential to perform a systematic evaluation based on clinical samples. We first evaluated the reliability of the above detection standard: we tested a single clinical sample at three different concentrations and measured each concentration in 20 replicates (Figure 2C). In our study, clinical samples of up to 1 × 10^4^ copies/ml could be detected with great accuracy by CDetection.v2. Meanwhile, we found that our system had good reproducibility. The specificity of the detection platform was further validated by testing other types of human coronaviruses and common viruses, as well as known viral nucleic acids that interfere with detection (Figure 2B and Supplementary Figure 4). The results of CDetection.v2 shall remain unaffected by the nucleic acid of other pathogens, medications taken by the patient, or interference from the patient themselves in the sample. That shown CDetection.v2 platform was able to sensitively detect SARS-CoV-2 without being affected by the presence of other compounds (Figure 2D). Taken together, the results indicate that CDetection.v2 has an impressive sensitivity, specificity, stability and sample inclusion in the detection of SARS-CoV-2.

### 3.3. The accuracy of CDetection.v2 is in agreement with that of RT-qPCR based on clinical samples

**FIGURE 3:**
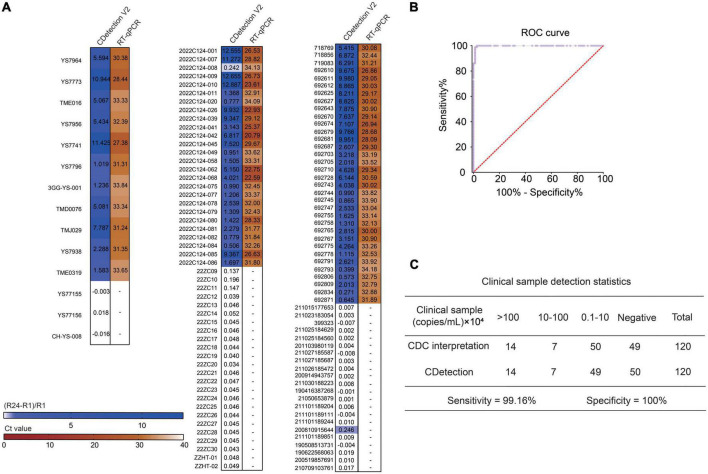
The accuracy of CDetection.v2 is in agreement with that of real-time quantitative PCR (RT-qPCR) based on clinical samples. **(A)** Clinical samples with known Ct values were detected by CDetection.v2. The (Rn24-Rn1)/Rn1 value is calculated in order to assess the conformity of the assay results. The results of the RT-qPCR are shown in brown, and CDetection.v2 in blue. **(B)** ROC curve analysis of CDetection.v2. **(C)** Statistical analysis of clinical samples. CDC, Center for Disease Control and Prevention.

The performance of detection system is determined by the sensitivity and specificity of the clinical samples detection. Therefore, we compared the sensitivity and specificity of CDetection.v2 with those of RT-qPCR by analyzing 120 cases of clinical samples (71 positive and 49 negative samples). These clinical samples were tested by CDetection.v2, and the sensitivity and specificity of CDetection.v2 were systematically evaluated by the (Rn24-Rn1)/Rn1 ratio. The (Rn24-Rn1)/Rn1 ratio and corresponding RT-qPCR results provided by the Centers for Disease Control and Prevention (CDC) in three different cities of China was compared. The heat map showed a high consistency between CDetection.v2 and RT-qPCR method (Figure 3A). The ROC curve of the outcomes of these two methods was further calculated. The plotted Receiver Operating Characteristic (ROC) curves showed that CDetection.v2 exhibited a specificity of 100% and a sensitivity of 99.16% (Figures 3B, C). Together, our newly developed one-step CDetection.v2 displayed the similar sensitivity and specificity as RT-qPCR on clinical sample detection.

### 3.4. Visualization of CDetection.v2 results

**FIGURE 4:**
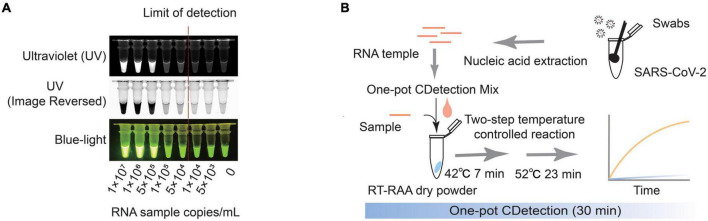
Visualization of CDetection.v2 results. **(A)** Visualization of CDetection.v2 results by exposing the samples under UV- or Blue-light. The RNA samples (5 × 10^3^, 1 × 10^4^, 5 × 10^4^, 1 × 10^5^) were all positive standard nucleic acid samples by gradient dilution, 0 copies/ml RNA sample is negative control. The dark red dotted line signifies the detection limit under visual detection. **(B)** Schematic diagram of the workflow of CDetection.v2.

In order to easily acquire the results by naked eyes which suit the specific situation such as customs, family and so forth, CDetection.v2 can be implemented without complicated instruments. Previous researches have utilized blue-light or ultraviolet to develop visual detection (Guo et al., 2020). The results of the CDetection.v2 platform can be directly assessed by exposing the tube containing the final reaction mixture to blue or ultraviolet light, and then interpreted directly by naked eyes. The positivity of samples with a concentration greater than 5 × 10^4^ copies/ml can be observed using either instrumentation or the naked eye (Figure 4A), indicating the great potential of CDetection.v2 for point of care testing (POCT). In summary, we have developed a Cas12b-assisted one-pot detection platform based on RT-RAA and CRISPR/Cas12b that provides rapid and accurate detection of SARS-CoV-2 within 30 min (Figure 4B).

## 4. Discussion

In this study, we developed a one-pot SARS-CoV-2 detection system, named as CDetection.v2 platform, which can detect standard SARS-CoV-2 samples with a sensitivity of 5,000 copies/ml within just 30 min. Results of 120 cases of COVID-19 throat swab samples detected by CDetection.v2 platform were completely in line with the results detected by RT-qPCR, indicating the high specificity and good compatibility of this platform for complex clinical samples. Apart from these advantages, CDetection.v2 platform also displayed several other advantages in SARS-CoV-2 detection. First of all, CDetection.v2 platform can detect most current SARS-CoV-2 variants. Since SARS-CoV-2 mutates rapidly and subsequently has given rise to various variants worldwide (Scovino et al., 2022), the universality of CDetection.v2 platform was assessed by comparing and analyzing the full sequence of SARS-CoV-2 by BLAST. A total of 3,548 sequences of SARS-CoV-2 were randomly selected from the NCBI database with a maximum of 100 samples per country and further classified based on region and time of identification. Statistical analysis of the mismatch sites in the SARS-CoV-2 sequence revealed that only a very small proportion of the sequences could not be detected by our platform. The RT-RAA-Rp primers used were able to detect nearly 99% of SARS-CoV-2, and the sgRNA was able to target 99.97% of SARS-CoV-2 sequences. To further verify the efficiency of CDetection.v2 platform in detecting different variants, the standard nucleic acid of the Delta and Omicron variants were diluted into a concentration of 1 × 10^4^ copies/ml, respectively, and further under tested by our platform. The results showed that CDetection.v2 platform could detect both variants (Supplementary Figure 5). Moreover, CDetection.v2 platform displays promising for POCT in the future, since we have tried to achieve the naked eye observed of the detection results. In comparison to our previous detection system, CDetection.v2 shows significantly improvement on detection speed and detection procedure without compromising its adequate accuracy (Supplementary Table 1).

Although the CDetection.v2 platform showed satisfactory sensitivity for the detection of SARS-CoV-2, in comparison to other current detection methods, optimizations on enhancing sensitivity are still needed (Supplementary Table 2). Additionally, the incompatibility between isothermal amplification and CRISPR is still needed to be addressed. To date, the reasons for this incompatibility have not been well understood, several factors might affect the compatibility of two steps. For examples, competitive binding of double-stranded DNA to Cas protein in both RT-RAA and CRISPR reaction system might affect the enzyme efficiency of Cas protein during CRISPR detection (Lu et al., 2022); single-stranded DNA-binding protein (SSB),as a critical component of RT-RAA, would not only bind to the RT-RAA primers, but also could bind to the sgRNA, resulting in the reduction of the effective concentration of sgRNA in the one-pot assay (Piepenburg et al., 2006; Routsias et al., 2010; Cook et al., 2018).

With continual advancement in the fields of electron microscopy and structural biology, a detailed analysis of the structures of a variety of Cas nucleases has been conducted, from the recognition of guide RNA, pre-processing, to the recognition and hydrolysis of target nucleic acids, including Cas12b (Yamano et al., 2016; Liu et al., 2017; Nishimasu et al., 2017; Xiao et al., 2021). Meanwhile, the mechanism of ssDNA hydrolysis also has been extensively explored. It has been demonstrated that Cas proteins can be modified to enhance nuclease activity (Chen et al., 2022; Mahas et al., 2022; Yang et al., 2022). Therefore, by utilizing rational design and directed evolution, the *trans*-cleavage activity of Cas12b, a component of the CRISPR/Cas12b system, can be further improved. On the other hand, the amplification step can also be a source of aerosols and false positives. Attention must be paid to the realization of an amplification-free, sensitive diagnostic platform based on the CRISPR system, as this will render the detection process more portable and cost-effective (Li et al., 2022). Efforts have been made within this field to integrate formats with enzyme cascade reactions or ddPCR (Liu et al., 2021; Shi et al., 2021; Xue et al., 2022). Merging multiple strategies to achieve engineering instant diagnosis *via* portable, accurate detection through the means of mini-instruments or later flow is a potential avenue for the future of this field (de Puig et al., 2021; Nguyen et al., 2021). Globally, infectious diseases such as Zika, HIV, smallpox, and Ebola virus display high mortality rates and pose a threat to human health. CRISPR/Cas12b-based systems will continue to progress and adapt to the changing needs and scenarios. Thus, the strategy underlying the CDetection.v2 system could also be used to detect these viruses and provide a sensitive, portable and rapid nucleic acid test for infectious diseases prevention and control.

## Data availability statement

The datasets presented in this study can be found in online repositories. The names of the repository/repositories and accession number(s) can be found in this article/Supplementary material.

## Ethics statement

The studies involving human participants were reviewed and approved by the Ethics Commission is QX-2022-001 the Ethics Committee of Hubei Provincial Center for Disease Control and Prevention (Hubei Academy of Preventive Medicine), 2021-73 (IRB for Preventive Medicine of Shandong Center for Disease Control and Prevention), and 2021-007-01 (the Ethics Committee for Drug Clinical Trials of Tonghua Central Hospital). Written informed consent to participate in this study was provided by the participants’ legal guardian/next of kin.

## Author contributions

WL, QZ, WP, and XW conceived the project and designed the experiments. XW, YC, WP, S-QW, and XC performed the experiments. WL, QZ, XW, YC, YZ, WP, XC, and YH analyzed the data. WL, QZ, YZ, XW, YC, YH, and S-QW wrote the manuscript. YF, RJ, KZ, TL, JW, KP, BL, JX, and YW provided clinical samples and ethics statements. All authors contributed to the article and approved the submitted version.

## Footnotes

1
https://www.ncbi.nlm.nih.gov/

2
https://ftp.ncbi.nlm.nih.gov/blast/executables/blast+/LATEST/
