# Supplementary material for: CDetection.v2: One-pot assay for the detection of SARS-CoV-2
Source: Front Microbiol. 2023 Mar 23;14:1158163. doi: 10.3389/fmicb.2023.1158163 (PMC10076661; doi:10.3389/fmicb.2023.1158163)
Supplement: Supplementary file 1 [file Data_Sheet_1.docx]

Supplementary Material

**CDetection.v2: One-pot assay for the detection of SARS-CoV-2**

**Xinge Wang^1234^†, Yangcan Chen^1234^†, Xuejia Cheng^5^†, Si-Qi Wang^123^†, Yanping Hu^1234^,** **Yingmei Feng^6^, Ronghua Jin^7^, Kangping Zhou^8^, Ti Liu^9^, Jianxing Wang^9^, Kai Pan^8^, Bing Liu^10^, Jie Xiang^11^, Yanping Wang^12^, Qi Zhou^1234^,Ying Zhang^123^, Weiye Pan^5^*, Wei Li^1234^***

*** Correspondence:** liwei@ioz.ac.cn; weiyepan@synsorbio.com.

# Supplementary Figures and Tables

## Supplementary Figures


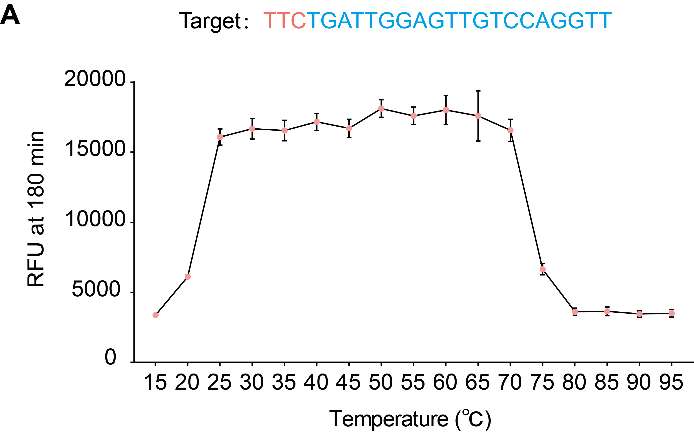


**Supplementary Figure 1.** **Comparison of Cas12b *trans*-cleavage activity at different temperatures**

(A) Comparison of the trans-cleavage activity of Cas12b at different temperatures. RFU, relative fluorescent unit. Error bars represent SD of mean, n = 3.

**
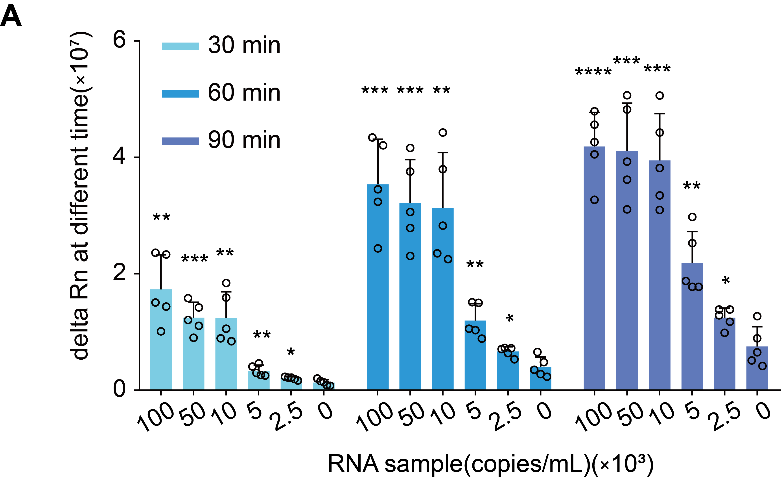
**

**Supplementary Figure 2.** **Analysis of the effect of detection time on sensitivity of CDetection.v2** (A) The sensitivity of CDetection.v2 was evaluated by delta Rn measured by RT-qPCR instrument, the RNA template was added into the system by gradient dilution. Error bars represent SD of mean, n = 5 biological replicates. Two-tailed, unpaired Student’s t test, **p < 0.05, **p < 0.01, ***p < 0.001, ****p < 0.0001.*


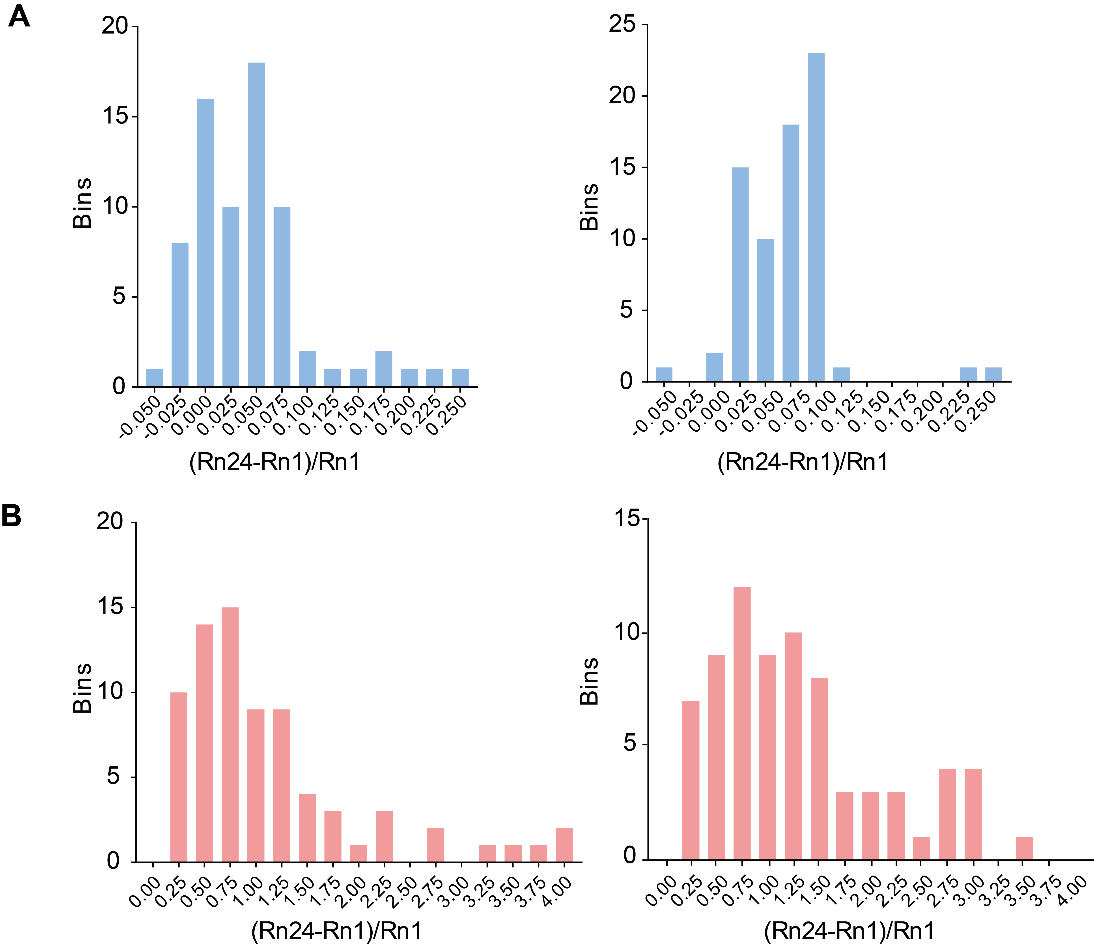


**Supplementary Figure 3.** **Determination of reference values for positive results**

(A) Determination of negative reference values for results interpretation. The detection reference values of different negative samples were analyzed. The assay efficiency is evaluated by calculating (Rn24-Rn1)/Rn1, Rn24 is the fluorescence signal value of cycle 24, Rn1 is the fluorescence signal value of cycle 1. (B) Determination of positive reference values for results interpretation. The detection reference values of different positive samples were analyzed. The assay efficiency is evaluated by (Rn24-Rn1)/Rn1, Rn24 is the fluorescence signal value of cycle 24, Rn1 is the fluorescence signal value of cycle 1.


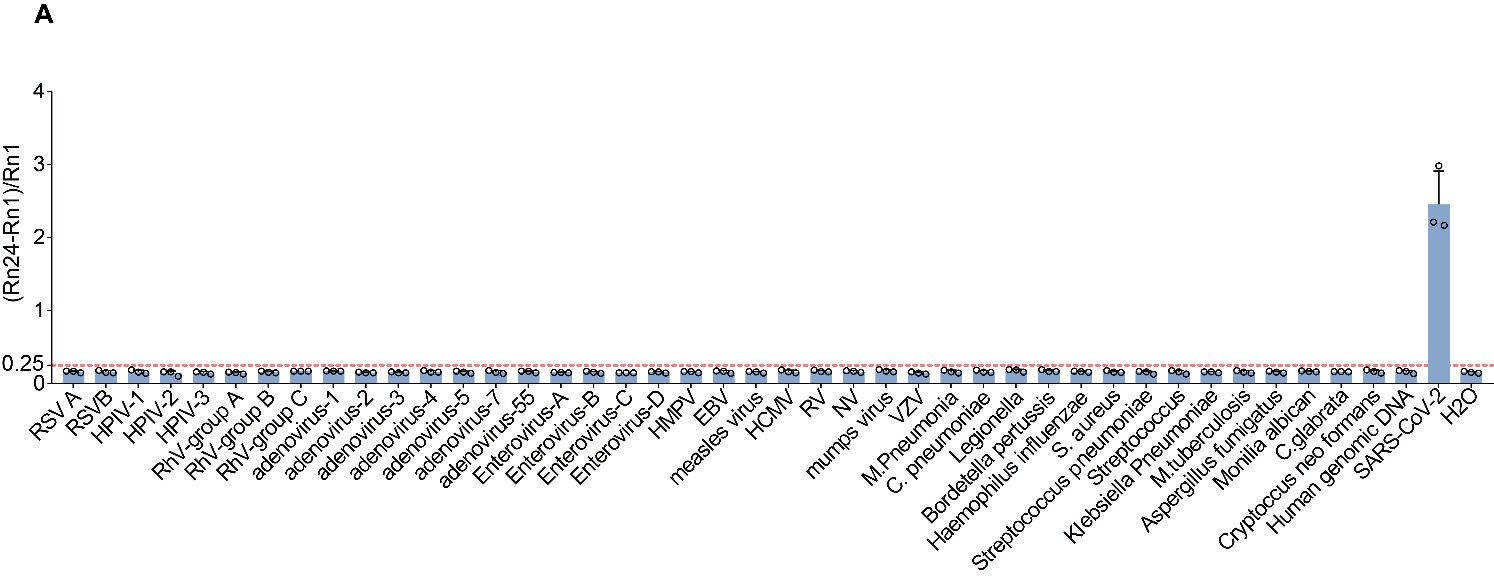


**Supplementary Figure 4.** **The analysis of specificity and commendable tolerance of clinical samples by CDetection.v2**

(A) Detection of other microbial nucleic acids commonly found in the respiratory system or nucleic acids commonly found in the environment. The value of (Rn24-Rn1)/Rn1 can determine the specificity of the CDetection.v2 system for SARS-CoV-2.


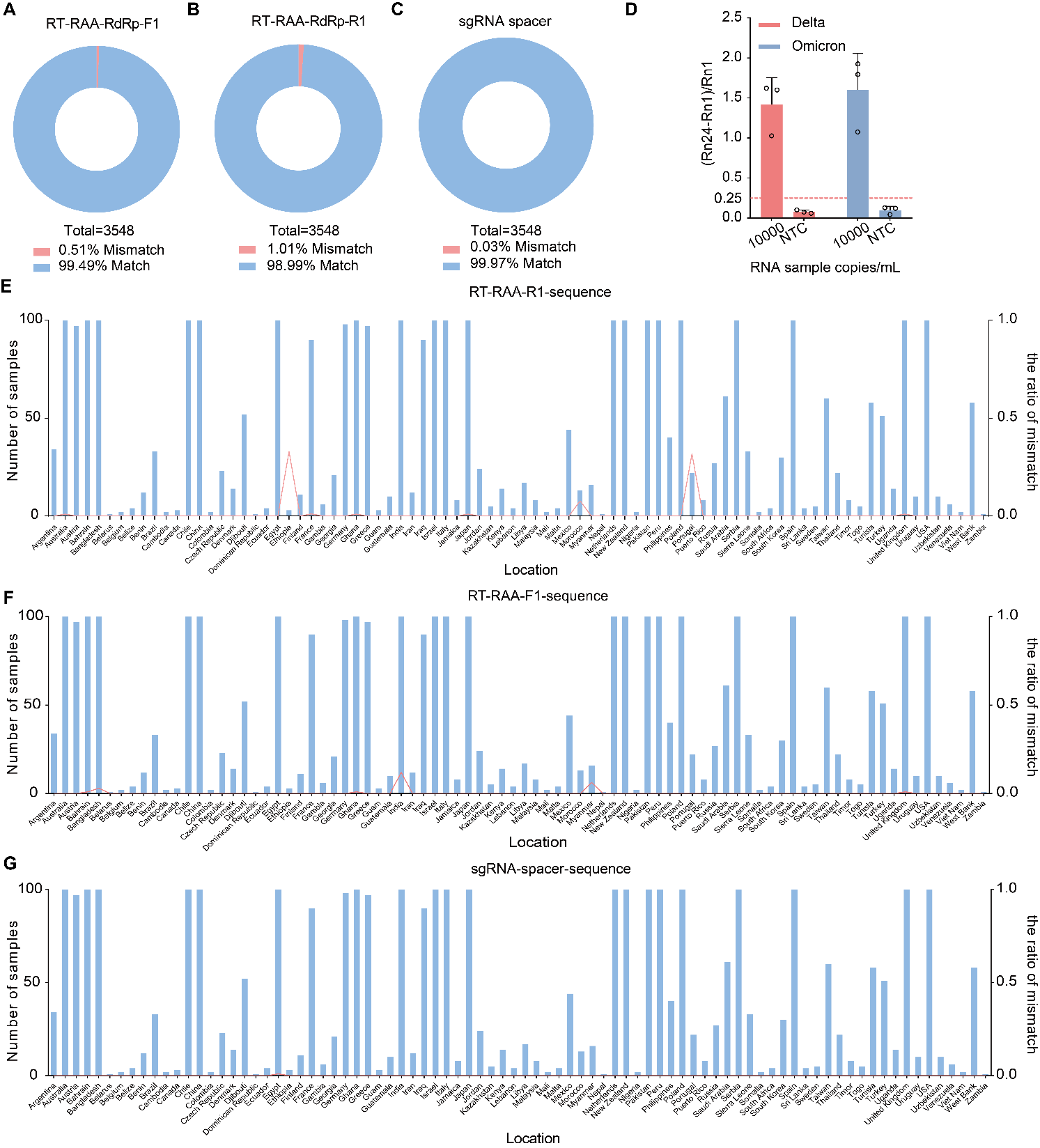


**Supplementary Figure 5.** **Analysis of sequence sample and regional inclusion**

(A) The pie chart was used to analyze the ratio of perfect match and mismatch of RT-RAA-RdRp-F1, sequences in SARS-COV-2 sequences. (B) The pie chart was used to analyze the ratio of perfect match and mismatch of RT-RAA-RdRp-R1 sequences in SARS-COV-2 sequences. (C) The pie chart was used to analyze the ratio of perfect match and mismatch of the spacer of sgRNA sequences in SARS-COV-2 sequences. (D) CDetection.v2 detects Delta and Omicron variants nucleic acids. The concentration of standard nucleic acid for Delta and Omicron variants were 10,000 copies / ml, (Rn24-Rn1)/Rn1, Rn24 is the fluorescence signal value of cycle 24, Rn1 is the fluorescence signal value of cycle 1. (E) The number of samples analyzed by the spacer of sgRNA in different regions and the proportion of mismatch sequences in them were analyzed. (F) The number of samples analyzed by RT-RAA-RdRp-F1 in different regions and the proportion of mismatch sequences in them were analyzed. (G) The number of samples analyzed by RdRp-RT-RAA-R1 in different regions and the proportion of mismatch sequences in them were analyzed.

## Supplementary Tables

**Supplementary Table 1. Comparation of CDetection.v2 with our previous works**

|  | CDetection | CASdetec | CDetection.v2 |
| --- | --- | --- | --- |
| Times of operation | Two-step | Two-step | One-step |
| Number of tubes needed | Two-tube | One-tube | One-tube |
| Reaction duration | 180 min | 60 min | 30 min |
| Specificity | High | High | High |
| Sensitivity | 1 åM | 5 × 10^3^ copies/ml | 5 × 10^3^ copies/ml |
| Type of target | DNA | RNA/DNA | RNA/DNA |
| The form of results | Fluorescence | UV/Fluorescence | UV/Fluorescence |
| Citation | (Teng et al., 2019) | (Guo et al., 2020) |  |

**Supplementary Table 2. Comparation of CDetection.v2 with other current detection methods**

|  | CDetection.v2 | SARS-CoV-2 DETECTR | STOPCovid.v2 |  |
| --- | --- | --- | --- | --- |
| Thermal cycling | No | No | No |  |
| Clinical sample size | 120 | 36 | 402 |  |
| Specificity | 100% | 100% | 98.50% |  |
| Sensitivity | 99.16% | 95% | 93.10% |  |
| One-pot | Yes | No | Yes |  |
| Assay components | 42 °C 7 min + 52 °C 23 min | 62 °C 20-30 min + 37 °C 10 min | 60 °C 45 min |  |
|  |  |  |  |  |
| Citation |  | (Broughton et al., 2020) | (Joung et al., 2020) |  |

**Supplementary Tables 3. Nucleic acid sequence used in this article**

| **FQ ssDNA reporter** | |
| --- | --- |
| polyT-FQ-7nt | FAM-TTTTTTT-BHQ1 |
| **DNA** | |
| Target_1_NTS-100 | AAACACTTACAGAAAGTTGTATTACCAGGTGGAAGGTTCTGATTGGAGTTGTCCAGGTTTTTGGCACGTTGAACAAATAATTGAACATCATGCATGAACA |
| Target_2_NTS-100 | CATTTTGCTGAATAAGCATATTGACGCATACAAAACATTCCCACCAACAGAGCCTAAAAAGGACAAAAAGAAGAAGGCTGATGAAACTCAAGCCTTACCG |
| ds_activator_RdRp_1(277bp) | GTTTATAGTGATGTAGAAAACCCTCACCTTATGGGTTGGGATTATCCTAAATGTGATAGAGCCATGCCTAACATGCTTAGAATTATGGCCTCACTTGTTCTTGCTCGCAAACATACAACGTGTTGTAGCTTGTCACACCGTTTCTATAGATTAGCTAATGAGTGTGCTCAAGTATTGAGTGAAATGGTCATGTGTGGCGGTTCACTATATGTTAAACCAGGTGGAACCTCATCAGGAGATGCCACAACTGCTTATGCTAATAGTGTTTTTAACATTT |
| T7-sgRNA-Target_1 | TAATACGACTCACTATAGGGTCTAAAGGACAGAATTTTTCAACGGGTGTGCCAATGGCCACTTTCCAGGTGGCAAAGCCCGTTGAACTTCAAGCGAAGTGGCACTGATTGGAGTTGTCCAGGTT |
| T7-sgRNA-Target_2 | TAATACGACTCACTATAGGGTCTAAAGGACAGAATTTTTCAACGGGTGTGCCAATGGCCACTTTCCAGGTGGCAAAGCCCGTTGAACTTCAAGCGAAGTGGCACAATGCACCGGGCTTACTTAA |
| T7-AasgRNA-RdRp-1 | TAATACGACTCACTATAGGGTCTAAAGGACAGAATTTTTCAACGGGTGTGCCAATGGCCACTTTCCAGGTGGCAAAGCCCGTTGAACTTCAAGCGAAGTGGCACTGGCATCTCCTGATGAGGTT |
| T7-AasgRNA-RdRp-2 | TAATACGACTCACTATAGGGTCTAAAGGACAGAATTTTTCAACGGGTGTGCCAATGGCCACTTTCCAGGTGGCAAAGCCCGTTGAACTTCAAGCGAAGTGGCACCCACCAACAGAGCCTAAAAA |
| T7-AasgRNA-RdRp-3 | TAATACGACTCACTATAGGGTCTAAAGGACAGAATTTTTCAACGGGTGTGCCAATGGCCACTTTCCAGGTGGCAAAGCCCGTTGAACTTCAAGCGAAGTGGCACACTCAATACTTGAGCACACT |
| **Primers** | |
| Target_1-F | AAACACTTACAGAAAGTTGTATTACCAGGT |
| Target_1-R | TGTTCATGCATGATGTTCAATTATTTGTTC |
| Target_2-F | CATTTTGCTGAATAAGCATATTGACGCATA |
| Target_2-R | CGGTAAGGCTTGAGTTTCATCAGCCTTCTT |
| RPA-RdRp-F1 | GTTGTAGCTTGTCACACCGTTTCTATAGATTAGC |
| RPA-RdRp-R1 | CCTGGTTTAACATATAGTGAACCGCCACACAT |
| T7-sgRNA-RdRp-3-R | AGTGTGCTCAAGTATTGAGTGTGCCACTTCGCTTGAAGTTCA |
| T7-sgRNA-target-1-R | AACCTGGACAACTCCAATCAGTGCCACTTCGCTTGAAGTTCA |
| T7-sgRNA-target-2-R | TTTTTAGGCTCTGTTGGTGGGTGCCACTTCGCTTGAAGTTCA |
| RdRP_SARSr-F3 | GARATGGTCATGTGTGGCGG |
| RdRP_SARSr-R2 | CTTGACARATGTTAAASACACTATTAGCATA |
| **Probe** | |
| RdRP_SARSr-P2 | FAM-CAGGTGGAACCTCATCAGGAGATGC-BHQ1 |
| **AasgRNA** | |
| AasgRNA-Target_1 | GUCUAAAGGACAGAAUUUUUCAACGGGUGUGCCAAUGGCCACUUUCCAGGUGGCAAAGCCCGUUGAACUUCAAGCGAAGUGGCACUGAUUGGAGUUGUCCAGGUU |
| AasgRNA-Target_2 | GUCUAAAGGACAGAAUUUUUCAACGGGUGUGCCAAUGGCCACUUUCCAGGUGGCAAAGCCCGUUGAACUUCAAGCGAAGUGGCACCCACCAACAGAGCCUAAAAA |
| AasgRNA-RdRp-3 | GUCUAAAGGACAGAAUUUUUCAACGGGUGUGCCAAUGGCCACUUUCCAGGUGGCAAAGCCCGUUGAACUUCAAGCGAAGUGGCACACUCAAUACUUGAGCACACU |
| **pEASY-Blunt plasmid** | |
| pEASY-Blunt-SARS-CoV-2-RdRp | ...ATATCTGCAGAATTGCCCTTGTTTATAGTGATGTAGAAAACCCTCACCTTATGGGTTGGGATTATCCTAAATGTGATAGAGCCATGCCTAACATGCTTAGAATTATGGCCTCACTTGTTCTTGCTCGCAAACATACAACGTGTTGTAGCTTGTCACACCGTTTCTATAGATTAGCTAATGAGTGTGCTCAAGTATTGAGTGAAATGGTCATGTGTGGCGGTTCACTATATGTTAAACCAGGTGGAACCTCATCAGGAGATGCCACAACTGCTTATGCTAATAGTGTTTTTAACATTTAAGGGCAATTCCAGCACACT |
| pEASY-Blunt-SARS-CoV-2-N | ...ATGTCTGATAATGGACCCCAAAATCAGCGAAATGCACCCCGCATTACGTTTGGTGGACCCTCAGATTCAACTGGCAGTAACCAGAATGGAGAACGCAGTGGGGCGCGATCAAAACAACGTCGGCCCCAAGGTTTACCCAATAATACTGCGTCTTGGTTCACCGCTCTCACTCAACATGGCAAGGAAGACCTTAAATTCCCTCGAGGACAAGGCGTTCCAATTAACACCAATAGCAGTCCAGATGACCAAATTGGCTACTACCGAAGAGCTACCAGACGAATTCGTGGTGGTGACGGTAAAATGAAAGATCTCAGTCCAAGATGGTATTTCTACTACCTAGGAACTGGGCCAGAAGCTGGACTTCCCTATGGTGCTAACAAAGACGGCATCATATGGGTTGCAACTGAGGGAGCCTTGAATACACCAAAAGATCACATTGGCACCCGCAATCCTGCTAACAATGCTGCAATCGTGCTACAACTTCCTCAAGGAACAACATTGCCAAAAGGCTTCTACGCAGAAGGGAGCAGAGGCGGCAGTCAAGCCTCTTCTCGTTCCTCATCACGTAGTCGCAACAGTTCAAGAAATTCAACTCCAGGCAGCAGTAGGGGAACTTCTCCTGCTAGAATGcGCTGGCAATGGCGGTGATGCTGCTCTTGCTTTGCTGCTGCTTGACAGATTGAACCAGCTTGAGAGCAAAATGTCTGGTAAAGGCCAACAACAACAAGGCCAAACTGTCACTAAGAAATCTGCTGCTGAGGCTTCTAAGAAGCCTCGGCAAAAACGTACTGCCACTAAAGCATACAATGTAACACAAGCTTTCGGCAGACGTGGTCCAGAACAAACCCAAGGAAATTTTGGGGACCAGGAACTAATCAGACAAGGAACTGATTACAAACATTGGCCGCAAATTGCACAATTTGCCCCCAGCGCTTCAGCGTTCTTCGGAATGTCGCGCATTGGCATGGAAGTCACACCTTCGGGAACGTGGTTGACCTACACAGGTGCCATCAAATTGGATGACAAAGATCCAAATTTCAAAGATCAAGTCATTTTGCTGAATAAGCATATTGACGCATACAAAACATTCCCACCAACAGAGCCTAAAAAGGACAAAAAGAAGAAGGCTGATGAAACTCAAGCCTTACCGCAGAGACAGAAGAAACAGCAAACTGTGACTCTTCTTCCTGCTGCAGATTTGGATGATTTCTCCAAACAATTGCAACAATCCATGAGCAGTGCTGACTCAACTCAGGCCTAA |
| **RNA** | |
| SARS-CoV-2-RdRp_RNA | GUUUAUAGUGAUGUAGAAAACCCUCACCUUAUGGGUUGGGAUUAUCCUAAAUGUGAUAGAGCCAUGCCUAACAUGCUUAGAAUUAUGGCCUCACUUGUUCUUGCUCGCAAACAUACAACGUGUUGUAGCUUGUCACACCGUUUCUAUAGAUUAGCUAAUGAGUGUGCUCAAGUAUUGAGUGAAAUGGUCAUGUGUGGCGGUUCACUAUAUGUUAAACCAGGUGGAACCUCAUCAGGAGAUGCCACAACUGCUUAUGCUAAUAGUGUUUUUAACAUUU |

**Supplementary Tables 2. Information of clinical samples used in this article** **(used in Figure 3)**

| Sample ID | Region | Gender | Age | Sampling time | Results | Sample concentration（copies/ml） |
| --- | --- | --- | --- | --- | --- | --- |
| YS7964 | Hubei | female | 66 | 2020 | positive | 1.4×10^4^ |
| YS7773 | Hubei | female | 60 | 2020 | positive | 5.3×10^4^ |
| TME016 | Hubei | female | 51 | 2020 | positive | 2.1×10^3^ |
| YS7956 | Hubei | male | 65 | 2020 | positive | 3.5×10^3^ |
| YS7741 | Hubei | male | 59 | 2020 | positive | 1.1×10^5^ |
| YS7796 | Hubei | female | 53 | 2020 | positive | 7.3×10^3^ |
| 3GG-YS-001 | Hubei | female | 27 | 2020 | positive | 1.3×10^3^ |
| TMD0076 | Hubei | female | 80 | 2020 | positive | 1.8×10^3^ |
| TMJ029 | Hubei | male | 31 | 2020 | positive | 7.7×10^3^ |
| YS7938 | Hubei | female | 68 | 2020 | positive | 7.1×10^3^ |
| TME0319 | Hubei | male | 38 | 2020 | positive | 1.4×10^3^ |
| YS77155 | Hubei | male | 54 | 2020 | negative | 0 |
| YS77156 | Hubei | male | 52 | 2020 | negative | 0 |
| CH-YS-008 | Hubei | male | 66 | 2020 | negative | 0 |
| Sample ID | Region | Gender | Age | Sampling time | Results | Sample concentration（copies/ml） |
| 2022C124-001 | Shandong | male | 40 | 2022 | positive | 2.0×10^5^ |
| 2022C124-007 | Shandong | male | 51 | 2022 | positive | 4.1×10^4^ |
| 2022C124-008 | Shandong | male | 40 | 2022 | positive | 1.0×10^3^ |
| 2022C124-009 | Shandong | male | 45 | 2022 | positive | 1.7×10^5^ |
| 2022C124-010 | Shandong | female | 42 | 2022 | positive | 1.5×10^6^ |
| 2022C124-011 | Shandong | female | 77 | 2022 | positive | 2.4×10^3^ |
| 2022C124-020 | Shandong | male | 66 | 2022 | positive | 1.1×10^3^ |
| 2022C124-026 | Shandong | female | 11 | 2022 | positive | 2.4×10^6^ |
| 2022C124-039 | Shandong | female | 47 | 2022 | positive | 3.3×10^4^ |
| 2022C124-041 | Shandong | male | 38 | 2022 | positive | 4.5×10^5^ |
| 2022C124-042 | Shandong | male | 21 | 2022 | positive | 1.1×10^7^ |
| 2022C124-045 | Shandong | male | 36 | 2022 | positive | 2.3×10^4^ |
| 2022C124-049 | Shandong | female | 45 | 2022 | positive | 1.5×10^3^ |
| 2022C124-058 | Shandong | male | 41 | 2022 | positive | 1.8×10^3^ |
| 2022C124-062 | Shandong | female | 53 | 2022 | positive | 2.8×10^6^ |
| Sample ID | Region | Gender | Age | Sampling time | Results | Sample concentration（copies/ml） |
| 2022C124-068 | Shandong | female | 60 | 2022 | positive | 3.1×10^6^ |
| 2022C124-075 | Shandong | female | 31 | 2022 | positive | 3.3×10^3^ |
| 2022C124-077 | Shandong | male | 10 | 2022 | positive | 1.7×10^3^ |
| 2022C124-078 | Shandong | male | 39 | 2022 | positive | 4.5×10^3^ |
| 2022C124-079 | Shandong | female | 51 | 2022 | positive | 3.4×10^3^ |
| 2022C124-080 | Shandong | female | 49 | 2022 | positive | 5.8×10^4^ |
| 2022C124-081 | Shandong | female | 35 | 2022 | positive | 5.3×10^3^ |
| 2022C124-082 | Shandong | male | 32 | 2022 | positive | 5.1×10^3^ |
| 2022C124-084 | Shandong | female | 51 | 2022 | positive | 3.8×10^3^ |
| 2022C124-085 | Shandong | female | 7 | 2022 | positive | 1.9×10^5^ |
| 2022C124-086 | Shandong | male | 36 | 2022 | positive | 5.2×10^3^ |
| 22ZC09 | Shandong | male | 28 | 2022 | negative | 0 |
| 22ZC10 | Shandong | female | 32 | 2022 | negative | 0 |
| 22ZC11 | Shandong | male | 23 | 2022 | negative | 0 |
| 22ZC12 | Shandong | female | 51 | 2022 | negative | 0 |
| Sample ID | Region | Gender | Age | Sampling time | Results | Sample concentration（copies/ml） |
| 22ZC13 | Shandong | female | 78 | 2022 | negative | 0 |
| 22ZC14 | Shandong | male | 83 | 2022 | negative | 0 |
| 22ZC15 | Shandong | female | 48 | 2022 | negative | 0 |
| 22ZC16 | Shandong | male | 19 | 2022 | negative | 0 |
| 22ZC17 | Shandong | male | 43 | 2022 | negative | 0 |
| 22ZC18 | Shandong | female | 39 | 2022 | negative | 0 |
| 22ZC19 | Shandong | male | 30 | 2022 | negative | 0 |
| 22ZC20 | Shandong | female | 18 | 2022 | negative | 0 |
| 22ZC21 | Shandong | male | 18 | 2022 | negative | 0 |
| 22ZC22 | Shandong | male | 20 | 2022 | negative | 0 |
| 22ZC23 | Shandong | male | 34 | 2022 | negative | 0 |
| 22ZC24 | Shandong | male | 74 | 2022 | negative | 0 |
| 22ZC25 | Shandong | male | 28 | 2022 | negative | 0 |
| 22ZC26 | Shandong | female | 81 | 2022 | negative | 0 |
| 22ZC27 | Shandong | male | 62 | 2022 | negative | 0 |
| Sample ID | Region | Gender | Age | Sampling time | Results | Sample concentration（copies/ml） |
| 22ZC28 | Shandong | female | 17 | 2022 | negative | 0 |
| 22ZC29 | Shandong | male | 72 | 2022 | negative | 0 |
| 22ZC30 | Shandong | male | 11 | 2022 | negative | 0 |
| ZZHT-01 | Shandong | female | 26 | 2022 | negative | 0 |
| ZZHT-02 | Shandong | female | 35 | 2022 | negative | 0 |
| 718769 | Jilin | male | 55 | 2022 | positive | 1.7×10^4^ |
| 718856 | Jilin | male | 55 | 2022 | positive | 3.3×10^3^ |
| 719083 | Jilin | male | 66 | 2022 | positive | 7.8×10^3^ |
| 692610 | Jilin | female | 60 | 2021 | positive | 1.6×10^5^ |
| 692611 | Jilin | male | 57 | 2021 | positive | 3.5×10^4^ |
| 692612 | Jilin | male | 70 | 2021 | positive | 1.8×10^4^ |
| 692625 | Jilin | female | 33 | 2021 | positive | 3.2×10^4^ |
| 692627 | Jilin | male | 43 | 2021 | positive | 1.8×10^4^ |
| 692643 | Jilin | male | 65 | 2021 | positive | 9.7×10^3^ |
| 692670 | Jilin | female | 26 | 2021 | positive | 3.3×10^4^ |
| Sample ID | Region | Gender | Age | Sampling time | Results | Sample concentration（copies/ml） |
| 692674 | Jilin | male | 54 | 2021 | positive | 1.5×10^5^ |
| 692679 | Jilin | female | 56 | 2021 | positive | 4.5×10^4^ |
| 692681 | Jilin | female | 51 | 2021 | positive | 6.8×10^4^ |
| 692687 | Jilin | male | 53 | 2021 | positive | 2.9×10^4^ |
| 692703 | Jilin | female | 52 | 2021 | positive | 2.0×10^3^ |
| 692705 | Jilin | female | 42 | 2021 | positive | 1.6×10^3^ |
| 692710 | Jilin | male | 68 | 2021 | positive | 2.9×10^4^ |
| 692728 | Jilin | female | 35 | 2021 | positive | 1.2×10^4^ |
| 692743 | Jilin | male | 27 | 2021 | positive | 1.8×10^4^ |
| 692744 | Jilin | female | 42 | 2021 | positive | 1.3×10^3^ |
| 692745 | Jilin | female | 7 | 2021 | positive | 1.2×10^3^ |
| 692747 | Jilin | female | 37 | 2021 | positive | 2.2×10^3^ |
| 692755 | Jilin | female | 42 | 2021 | positive | 2.1×10^3^ |
| 692758 | Jilin | female | 64 | 2021 | positive | 4.1×10^3^ |
| 692765 | Jilin | male | 73 | 2021 | positive | 1.8×10^4^ |
| Sample ID | Region | Gender | Age | Sampling time | Results | Sample concentration（copies/ml） |
| 692767 | Jilin | male | 18 | 2021 | positive | 9.7×10^3^ |
| 692775 | Jilin | male | 33 | 2021 | positive | 1.9×10^3^ |
| 692778 | Jilin | female | 80 | 2021 | positive | 3.1×10^3^ |
| 692791 | Jilin | female | 26 | 2021 | positive | 1.2×10^3^ |
| 692793 | Jilin | male | 15 | 2021 | positive | 1.0×10^3^ |
| 692806 | Jilin | male | 26 | 2021 | positive | 2.7×10^3^ |
| 692809 | Jilin | male | 71 | 2021 | positive | 2.6×10^3^ |
| 692834 | Jilin | male | 19 | 2021 | positive | 2.5×10^3^ |
| 692871 | Jilin | male | 22 | 2021 | positive | 4.9×10^3^ |
| 211015177653 | Jilin | female | 32 | 2021 | negative | 0 |
| 211023183054 | Jilin | male | 30 | 2021 | negative | 0 |
| 399323 | Jilin | male | 42 | 2021 | negative | 0 |
| 211025184629 | Jilin | female | 22 | 2021 | negative | 0 |
| 211025184560 | Jilin | male | 22 | 2021 | negative | 0 |
| 201103980119 | Jilin | male | 66 | 2021 | negative | 0 |
| Sample ID | Region | Gender | Age | Sampling time | Results | Sample concentration（copies/ml） |
| 211027185587 | Jilin | male | 27 | 2021 | negative | 0 |
| 211027185687 | Jilin | male | 31 | 2021 | negative | 0 |
| 211026185472 | Jilin | male | 29 | 2021 | negative | 0 |
| 200914943757 | Jilin | male | 66 | 2021 | negative | 0 |
| 211030188223 | Jilin | male | 47 | 2021 | negative | 0 |
| 190416387268 | Jilin | male | 32 | 2021 | negative | 0 |
| 21050653879 | Jilin | female | 34 | 2021 | negative | 0 |
| 211101189204 | Jilin | male | 40 | 2021 | negative | 0 |
| 211101189111 | Jilin | female | 69 | 2021 | negative | 0 |
| 211101189244 | Jilin | female | 36 | 2021 | negative | 0 |
| 200810915644 | Jilin | male | 53 | 2021 | negative | 0 |
| 211101189851 | Jilin | female | 20 | 2021 | negative | 0 |
| 190508513731 | Jilin | female | 61 | 2021 | negative | 0 |
| 190622568063 | Jilin | female | 60 | 2021 | negative | 0 |
| 200519857691 | Jilin | female | 54 | 2021 | negative | 0 |
| 210709103761 | Jilin | male | 71 | 2021 | negative | 0 |

**Information of clinical samples used in used in Supplementary Figure 2**

| Sample ID | Region | Gender | Age | Sampling time | Results | Sample concentration（copies/mL） |
| --- | --- | --- | --- | --- | --- | --- |
| YS7800 | Hubei | male | 83 | 2020 | positive | 1.9×10^4^ |
| TMB0004 | Hubei | male | 40 | 2020 | positive | 3.0×10^4^ |
| 3GG-YS-003 | Hubei | female | 65 | 2020 | positive | 2.7×10^4^ |
| 3GG-YS-004 | Hubei | female | 35 | 2020 | positive | 8.3×10^4^ |
| 3GG-YS-009 | Hubei | male | 70 | 2020 | positive | 1.8×10^4^ |
| 2022C124-008 | Shandong | male | 40 | 2022 | positive | 6.3×10^4^ |
| 2022C124-028 | Shandong | male | 34 | 2022 | positive | 3.3×10^6^ |
| 2022C124-061 | Shandong | female | 48 | 2022 | positive | 1.4×10^5^ |
| 2022C124-068 | Shandong | female | 60 | 2022 | positive | 2.1×10^5^ |
| 2022C124-084 | Shandong | female | 51 | 2022 | positive | 3.3×10^5^ |
| 718782 | Jilin | male | 38 | 2022 | positive | 4.9×10^4^ |
| 718742 | Jilin | male | 33 | 2022 | positive | 4.3×10^4^ |
| 718773 | Jilin | female | 50 | 2022 | positive | 5.1×10^4^ |
| 718776 | Jilin | male | 54 | 2022 | positive | 1.9×10^5^ |
| Sample ID | Region | Gender | Age | Sampling time | Results | Sample concentration（copies/mL） |
| 719019 | Jilin | female | 16 | 2022 | positive | 1.2×10^5^ |
| FK-YS-016 | Hubei | female | 36 | 2020 | negative | 0 |
| FK-YS-026 | Hubei | male | 33 | 2020 | negative | 0 |
| YS7077 | Hubei | female | 55 | 2020 | negative | 0 |
| CH-YS-007 | Hubei | female | 64 | 2020 | negative | 0 |
| 22ZC05 | Shandong | female | 21 | 2022 | negative | 0 |
| 22ZC06 | Shandong | male | 58 | 2022 | negative | 0 |
| 22ZC07 | Shandong | male | 14 | 2022 | negative | 0 |
| 22ZC08 | Shandong | male | 36 | 2022 | negative | 0 |
| 191207744080 | Jilin | female | 68 | 2021 | negative | 0 |
| 201109984619 | Jilin | female | 67 | 2021 | negative | 0 |
| 21060275274 | Jilin | female | 52 | 2021 | negative | 0 |
| 20120810122 | Jilin | male | 32 | 2021 | negative | 0 |

**References**

Broughton, J.P., Deng, X., Yu, G., Fasching, C.L., Servellita, V., Singh, J., Miao, X., Streithorst, J.A., Granados, A., Sotomayor-Gonzalez, A.*, et al.* (2020). CRISPR-Cas12-based detection of SARS-CoV-2. Nat Biotechnol *38*, 870-874.

Guo, L., Sun, X., Wang, X., Liang, C., Jiang, H., Gao, Q., Dai, M., Qu, B., Fang, S., Mao, Y.*, et al.* (2020). SARS-CoV-2 detection with CRISPR diagnostics. Cell Discov *6*, 34.

Joung, J., Ladha, A., Saito, M., Kim, N.G., Woolley, A.E., Segel, M., Barretto, R.P.J., Ranu, A., Macrae, R.K., Faure, G.*, et al.* (2020). Detection of SARS-CoV-2 with SHERLOCK One-Pot Testing. N Engl J Med.

Teng, F., Guo, L., Cui, T., Wang, X.G., Xu, K., Gao, Q., Zhou, Q., and Li, W. (2019). CDetection: CRISPR-Cas12b-based DNA detection with sub-attomolar sensitivity and single-base specificity. Genome Biol *20*, 132.
